# Experimental Study and Early Clinical Application Of a Sutureless Aortic Bioprosthesis

**DOI:** 10.5935/1678-9741.20150072

**Published:** 2015

**Authors:** Walter J. Gomes, João Carlos Leal, Fabio Biscegli Jatene, Nelson A. Hossne Jr, Renata Gabaldi, Glaucia Basso Frazzato, Guilherme Agreli, Domingo M. Braile

**Affiliations:** 1 Escola Paulista de Medicina da Universidade Federal de São Paulo (EPM-UNIFESP), São Paulo, SP, Brazil.; 2 Sociedade Portuguesa de Beneficência de São José do Rio Preto, SP, Brazil.; 3 Instituto do Coração do Hospital das Clínicas da Faculdade de Medicina da Universidade de São Paulo (InCor HC-FMUSP), São Paulo, SP, Brazil.; 4 Escola Paulista de Medicina da Universidade Federal de São Paulo (EPM-UNIFESP), São Paulo, SP, Brazil and Hospital São Paulo (HSP), São Paulo, SP, Brazil.; 5 Braile Biomédica, São José do Rio Preto, SP, Brazil.; 6 Faculdade de Medicina de São José do Rio Preto (FAMERP), São José do Rio Preto, SP, Brazil.

**Keywords:** Aortic Valve, Surgery, Heart Valves, Surgery, Aortic Valve Stenosis, Bioprosthesis

## Abstract

**INTRODUCTION:**

The conventional aortic valve replacement is the treatment of choice for symptomatic severe aortic stenosis. Transcatheter technique is a viable alternative with promising results for inoperable patients. Sutureless bioprostheses have shown benefits in high-risk patients, such as reduction of aortic clamping and cardiopulmonary bypass, decreasing risks and adverse effects.

**OBJECTIVE:**

The objective of this study was to experimentally evaluate the implantation of a novel balloon-expandable aortic valve with sutureless bioprosthesis in sheep and report the early clinical application.

**METHODS:**

The bioprosthesis is made of a metal frame and bovine pericardium leaflets, encapsulated in a catheter. The animals underwent left thoracotomy and the cardiopulmonary bypass was established. The sutureless bioprosthesis was deployed to the aortic valve, with 1/3 of the structure on the left ventricular face. Cardiopulmonary bypass, aortic clamping and deployment times were recorded. Echocardiograms were performed before, during and after the surgery. The bioprosthesis was initially implanted in an 85 year-old patient with aortic stenosis and high risk for conventional surgery, EuroSCORE 40 and multiple comorbidities.

**RESULTS:**

The sutureless bioprosthesis was rapidly deployed (50-170 seconds; average=95 seconds). The aortic clamping time ranged from 6-10 minutes, average of 7 minutes; the mean cardiopulmonary bypass time was 71 minutes. Bioprostheses were properly positioned without perivalvar leak. In the first operated patient the aortic clamp time was 39 minutes and the patient had good postoperative course.

**CONCLUSION:**

The deployment of the sutureless bioprosthesis was safe and effective, thereby representing a new alternative to conventional surgery or transcatheter in moderate- to high-risk patients with severe aortic stenosis.

**Table:** 

**Abbreviations, acronyms & symbols**	
AVR	= Aortic valve replacement
CABG	= Coronary artery bypass grafting
COBEA	= Brazilian College of Animal Experimentation
CPB	= Cardiopulmonary bypass
TAVI	= Transcatheter technique

## INTRODUCTION

Conventional aortic valve replacement is still the treatment of choice for patients with symptomatic severe aortic valve stenosis. However, in recent times the transcatheter technique (TAVI) has emerged as a viable and effective alternative to treat high risk or inoperable patients^[1]^.

Nevertheless, inherent complications of TAVI has been surfacing and restricting its use, such as the embolization of calcium debris and consequent cerebral infarction, peripheral vascular damage, the further need of pacemaker insertion, paravalvular leakage and its impact on long-term survival, coronary ostium occlusion, aortic rupture and the high cost of the device^[2,3]^.

Sutureless AVR using self-expanding bioprosthesis is a new and promising alternative to standard AVR in elderly and high-risk surgical patients^[4]^. The proposed benefits of this technology include enhanced implantability, shorter aortic cross-clamp and cardiopulmonary bypass (CPB) times, favourable hemodynamic performance, and easier access for minimally invasive surgery^[5-8]^. In addition, this approach allows complete removal of the diseased native valve and also comprises a suitable alternative to multiple valve procedures or associated coronary artery bypass grafting. Several European case series have shown excellent early clinical and hemodynamic outcomes^[7,8]^. Therefore sutureless aortic bioprostheses has been placed as an alternative to standard surgical AVR or TAVI in elderly and high-risk patients.

Comparative reports in intermediate- to high-risk patients have demonstrate a lower rate of perioperative complications and improved survival at 24-month follow-up with sutureless valves compared to TAVI^[2,9]^.

Therefore the objective of this study was to experimentally evaluate the implantation of a novel balloon-expandable aortic valve with sutureless bioprosthesis in animal model and report the early clinical application.

## METHODS

**Fig. 1:**
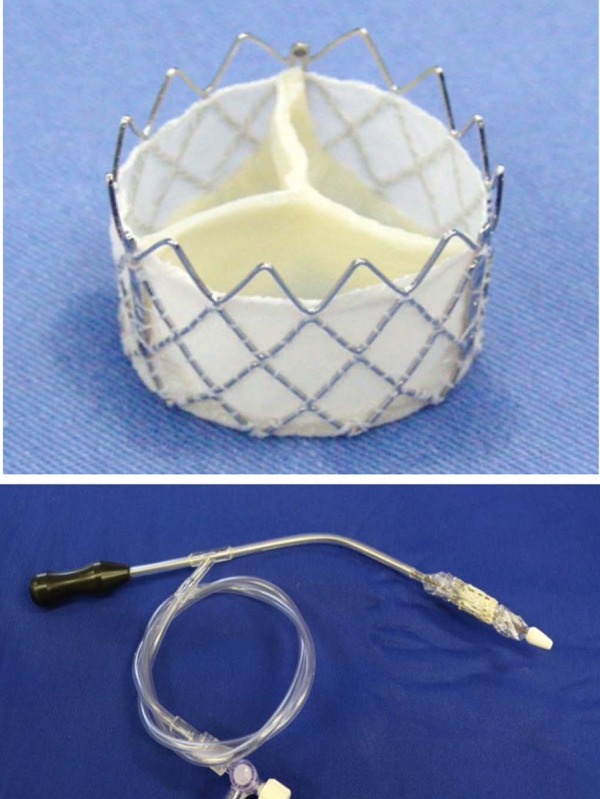
The Inovare Alpha sutureless bioprosthesis (above) and the delivery catheter (below).

The Inovare Alpha bioprosthesis is made of a metallic structure of cobalt-chrome, previously coated with a polyester fabric. This structure serves as a support for a bovine pericardium valve, which is sutured with polyester yarn to this metal support (Figure 1). The bioprosthesis is encapsulated in a catheter for the positioning and deployment. The fixing of the valve to the patient is given by the radial expansion force to the metal structure exerts against the patient valve structures, pressure sufficient enough to counterbalance the force exerted by blood flow.

The Inovare Alpha was evaluated in five animals (ovine) operated on in an experimental operative room under routine hemodynamic monitoring and conducted as usual in clinical cardiac surgical practice. The study was approved by the Institutional Ethics Committee and all animals were treated according to ethical principles of "National Research Council - Institute of Laboratory Animal Resources" and those drawn-up by the Brazilian College of Animal Experimentation (COBEA), along with the local Ethics Committee on Animal Use.

Standard general anesthesia and endotracheal intubation were applied for all surgical interventions. The animals underwent left thoracotomy and upon opening the pericardium the cardiopulmonary bypass was established through cannulation of carotid artery and left jugular vein. After aortic cross-clamping, myocardial protection was achieved using intermittent cold blood cardioplegia. Transverse aortotomy well above the aortic annulus enabled access to the aortic valve, the leaflets were removed and the sutureless bioprosthesis was balloon expanded and deployed to the aortic annulus, with 1/3 of the structure remaining on the left ventricular face. CPB, aortic clamping and deployment times were recorded. Echocardiograms were performed before, during and after the surgery. The initial clinical application was performed in an 85 year-old patient with aortic stenosis and high risk for conventional surgery, EuroSCORE 40% and multiple comorbidities associated with active hepatitis C.

## RESULTS

**Fig. 2:**
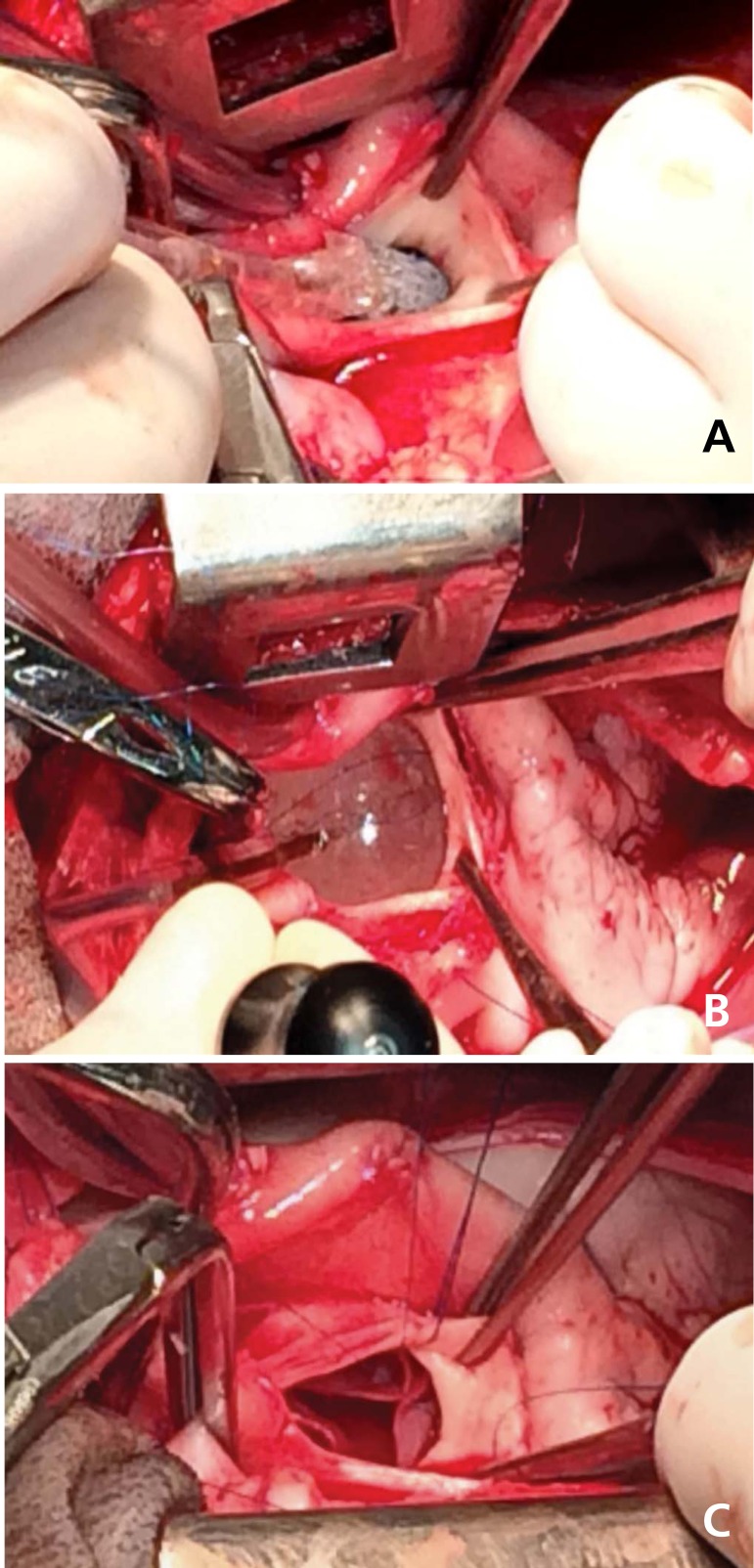
The deployment of the Inovare-Alpha in the experimental setting. A=transverse aortotomy well above the aortic annulus and insertion of the catheter-mounted valve; B=balloon expansion of the prosthesis; C=valve deployed to the aortic annulus

Valve deployment was successfully performed in all cases. All valves were firmly positioned without any migration. The sutureless bioprosthesis were rapidly deployed (time ranging from 50 to 170 seconds; average: 95 seconds). The aortic clamping time varied from 6-10 minutes, average of 7 minutes; the mean CPB time was 71 minutes. Bioprostheses were properly positioned and secured to the aortic ring, as assessed by transesophageal echo (Figure 2). There were neither paravalvular nor transvalvular leaks and excellent hemodynamic function was observed in all cases. All coronary arteries remained patent, with no obstruction determined by the device. Positioning and function were confirmed by autopsy in all but one animal.

In the postmortem examination, macroscopic examination revealed that all valves were fully deployed and expanded, and there was no obstruction of coronary ostia in any of the cases. The sutureless valves showed precise positioning in all cases with good alignment to the aortic valve plane.

**Fig. 3:**
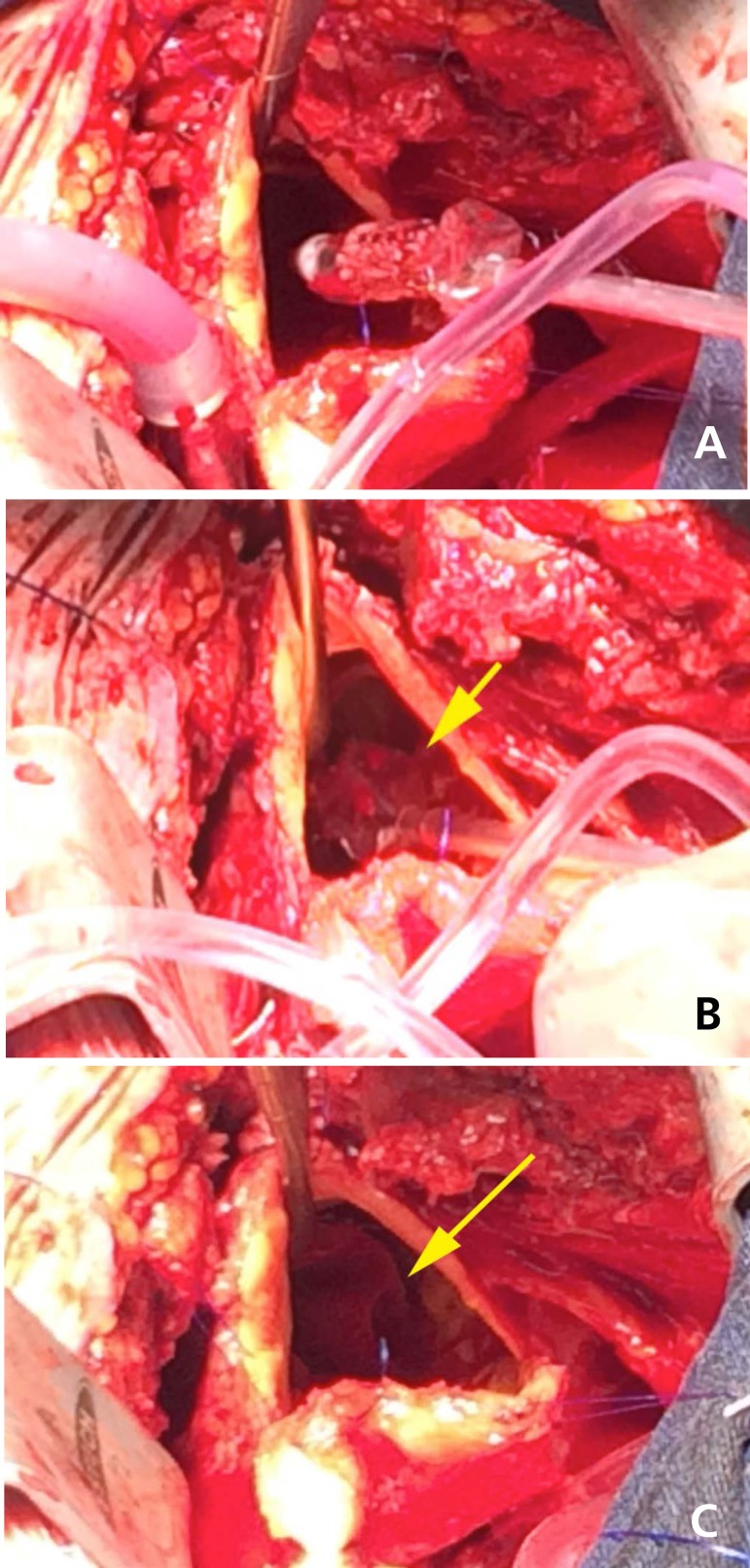
The clinical case.

In the first patient operated on, the prosthesis was inserted and the aortic clamping time was 39 minutes (Figure 3). The patient had a good postoperative recovery and has currently been followed up for the hepatitis C.

## DISCUSSION

The present study demonstrates that the implanted sutureless prosthesis proved to be reliable and efficient, sitting and remaining well attached to the aortic valve annulus with a fast procedure, as demonstrated by the short clamping time. The performance of the prosthesis was also consistent, without paravalvar leakage, migration, or damage to the surrounding tissues. These findings were confirmed by the postmortem examination.

A good alignment of the device and the aortic valve plane was observed, and a fair hemodynamic performance can be inferred because of the low profile and the optimized opening area. No interference to the coronary arteries was seen, with the metallic frame staying away from both ostia.

Surgical aortic valve replacement (AVR) still represents the gold standard among the therapeutical options in patients with severe aortic valve stenosis^[10]^.

Nevertheless, over the past few years, the possibility to treat high-risk or inoperable patients with alternative approaches, such as the transcatheter technique came out as a feasible and effective strategy with promising results. However, inherent complications of this new technology as its increased costs, the lack of removal of the calcified aortic valve and the resultant risk of paravalvular leakage, coronary occlusion and aortic rupture have been recognized as important limitations for TAVI^[11-13]^. For these reasons, a number of sutureless aortic valve bioprostheses have been developed to facilitate AVR and reduce the duration of aortic cross-clamping time and its related adverse events^[14]^.

The introduction of balloon-expandable sutureless bioprosthesis represents a step forward and a novel device for treating intermediate- to high-risk patients with severe aortic stenosis, with the reduction of aortic clamping and cardiopulmonary bypass (CPB), decreasing risks and adverse effects, also comprising a suitable alternative to multiple valve procedures or associated coronary artery bypass grafting. In addition, this approach allows complete or selective removal of the calcified and diseased native valve, potentially averting particulated cerebral embolism and cerebrovascular accident.

The concept of sutureless prosthetic heart valves led to the development of an array of new generation of devices. Nowadays, three sutureless aortic bioprostheses are currently available in Europe, the Perceval S (Sorin Group, Saluggia, Italy), the 3f Enable (Medtronic, Minneapolis, MN, USA), and the Intuity (Edwards Lifesciences, Irvine, CA, USA)^[14]^.

This novel surgical prosthesis has been favourably compared with the TAVI approach in recent series, thus offering a potential alternative to transcatheter in high-risk patients. Several European case series have shown good outcomes of sutureless compared to TAVI, with rather lower incidence of significant paravalvular regurgitation, post-procedural pacemaker implantation and peripheral vascular complications, along with better immediate postoperative survival^[2,9,10,12]^.

The potential of shortening surgical times and improving overall patient outcomes may expand the applicability of this simple and rapid implantation technique, as in long and complex procedures (reoperations or combined procedures). Reduced implantation and cross-clamping times will have a positive impact on the postoperative outcome of high-risk patients undergoing long surgical procedures^[5,6]^. Ranucci et al.^[15]^ reported that the aortic cross-clamp time is an independent predictor of severe cardiovascular morbidity, with an increased risk of 1.4% per 1-minute increase.

Associated with minimally invasive AVR, the sutureless approach can combine the advantages of both techniques, as demonstrated by several recently published case series that have shown excellent clinical and hemodynamic results^[16-18]^.

Additionally, it represents a formidable alternative for valve in valve (aortic or mitral), not only with failed bioprosthesis but also with mechanical valves, where the direct approach allows the disk removal and the rapid insertion of the sutureless valve.

Sutureless AVR is also an appealing option in several other specific circumstances, such as redo procedures, as well as in the presence of porcelain aorta, calcified aortic homograft, or small aortic annulus^[19-22]^. And the additional breakthrough is the performance of these procedures without the need of a hybrid room or a cath lab, being routinely carried out in an ordinary operative room simply with the aid of a transesophageal echo.

Consequently, the costs of sutureless are believed to be lower, as the price of TAVI devices are higher and requires incremental costs related to prosthesis implantation-related technology and to an increased number of personnel involved in this procedure. A cost-utility analysis of TAVI in Belgium concluded that it is not recommended to reimburse TAVI for high-risk patients because the patients had no survival benefit after 1 year, the risk of cerebrovascular accident was twice as high, and the costs were significantly higher^[23,24]^.

Definitely further prospective clinical trials are needed to determine the long-term durability and outcomes. The clinical trial testing these devices has been approved and is currently underway.

## CONCLUSION

In conclusion, the deployment of the sutureless bioprosthesis was safe and effective, thereby representing a new alternative to conventional surgery or transcatheter in moderate- to high-risk patients with severe aortic stenosis.

**Table:** 

**Authors' roles & responsibilities**	
WJG	Analysis/interpretation of data; final manuscript approval; study design; implementation of projects/ experiments; manuscript writing or critical review of its content
JCL	Analysis/interpretation of data; final manuscript approval; study design; implementation of projects/ experiments; manuscript writing or critical review of its content
FBJ	Final manuscript approval; study design; manuscript writing or critical review of its content
NAHJ	Analysis/interpretation of data; final manuscript approval; study design; manuscript writing or critical review of its content
RG	Conception and design study; final manuscript approval; manuscript writing or critical review of its content; conducted operations and/or trials
GBF	Analysis and/or data interpretation; conception and design study; final manuscript approval; manuscript writing or critical review of its content; conducted operations and/or trials
GA	Analysis and/or data interpretation; conception and design study; final manuscript approval; manuscript writing or critical review of its content; conducted operations and/or trials
DMB	Conception and design study; manuscript writing or critical review of its content; realization of operations and/or trials; final manuscript approval
